# Heavy metal contamination and blue carbon sequestration in mangrove ecosystems of Puerto Rico

**DOI:** 10.1002/jeq2.70078

**Published:** 2025-08-30

**Authors:** Jahnelle Howe, Peter M. Groffman, William J. Hernández, Shakila Merchant

**Affiliations:** ^1^ Department Earth and Environmental Sciences City University of New York, Graduate Center New York New York USA; ^2^ City University of New York, Advanced Science Research Center at The Graduate Center New York New York USA; ^3^ Research and Development Center University of Puerto Rico Mayagüez Puerto Rico; ^4^ City University of New York, Remote Sensing Earth System (CREST) Institute New York New York USA

## Abstract

Heavy metal contamination in coastal ecosystems can significantly impact biological activity, metal retranslocation, and biogeochemical cycling. This study assessed the concentrations of arsenic (As), cadmium (Cd), chromium (Cr), copper (Cu), nickel (Ni), lead (Pb), and zinc (Zn) in mangrove sediments and leaves of two ecosystems in Puerto Rico that differed in their proximity to urban areas: La Parguera and Laguna Grande. Metal bioconcentration factors and retranslocation percentages (RT%) were determined. Relationships between metals, between metals and sediment carbon, and metal retranslocation and bioavailability differed between the sites. Metals with high retranslocation percentages by plants, such as zinc and lead at La Parguera, suggest that plant‐mediated stabilization processes can reduce immediate bioavailability but may pose latent risks under changing environmental conditions. Conversely, cadmium, with low retranslocation, and nickel, with high retranslocation and high bioavailability at Laguna Grande, indicate greater potential for biological uptake and ecosystem stress. Results suggest that differences in relationships between metals and between metals and carbon may help identify sources and effects of metals. Further research is needed to explore the direct physiological effects of metal exposure on plants and their implications for carbon storage and ecosystem health in mangrove‐dominated systems.

## Core Ideas

- Metal levels vary due to anthropogenic inputs (Laguna Grande) and tidal flushing (La Parguera).
- This study broadens known metal concentration ranges in mangrove sediments for future research.
- Metal‐carbon correlations differ by site, serving as indicators of metal sources and conditions.
- Metal retranslocation and bioavailability depend on type and site conditions, posing potential risks.
- Site‐specific factors regulate metals, highlighting the need for targeted mangrove management strategies.

## Plain Language Summary

Mangrove forests help protect coastlines and store carbon, but they can also trap pollutants like heavy metals. This study examined the levels of arsenic (As), cadmium (Cd), chromium (Cr), copper (Cu), nickel (Ni), lead (Pb), and zinc (Zn) in the sediments and leaves of two Puerto Rican mangrove ecosystems: La Parguera and Laguna Grande. We found that metal levels were generally low, but Laguna Grande had higher levels due to human activity. Metals behaved differently at each site, with some binding to sediment carbon and others being taken up by plants. Some metals, like zinc and lead, were held by plants, reducing immediate risks but potentially causing future problems. Others, like cadmium and nickel, were more available, increasing the chance of harm to plants and animals. These results show that local environmental factors affect how metals move and impact ecosystems, helping scientists and policymakers monitor mangrove health.

**Abbreviations**
BCF: bioconcentration factor
GL: green leaves
RT%: retranslocation percentages
SL: senescent leaves

## 1 INTRODUCTION

Mangrove forests are vital components of tropical and subtropical coastlines, functioning as critical interfaces between land and sea (Alongi, 2002; Donato et al., 2011). They provide essential habitat and nursery grounds for numerous fish and invertebrate species, supporting both biodiversity and fisheries productivity (Nagelkerken et al., 2008). In addition to their ecological roles, mangroves provide essential ecosystem services by stabilizing shorelines, reducing coastal erosion, and attenuating wave energy, thereby enhancing coastal protection and resilience to extreme weather events (Mazda et al., 2006; Menéndez et al., 2020). Despite these benefits, mangroves are under severe pressure from anthropogenic activities such as coastal development, aquaculture, and deforestation, leading to widespread degradation and loss globally (Friess et al., 2019; Valiela et al., 2001).

Mangrove ecosystems are increasingly threatened by heavy metal (HM) contamination from industrial activities, urbanization, and agricultural runoff. In Puerto Rico, elevated levels of lead (Pb), mercury (Hg), cadmium (Cd), and zinc (Zn) have been found in mangrove sediments and tissues across multiple sites (Xiao et al., 2021). These levels are especially high in areas near industrial and urban sites, where improper waste disposal and untreated sewage are significant sources of pollution (Bashir et al., 2020). HMs can impair mangrove growth, reduce physiological function, and diminish ecosystem productivity (Nguyen et al., 2020). They persist in sediments, threatening biodiversity and bioaccumulating through food webs—from invertebrates to fish and birds (Khotimah et al., 2024). Monitoring and mitigation are crucial for protecting Puerto Rico's mangroves and their associated ecological services.

Mangrove growth sequesters blue carbon (C) through the process of photosynthesis. In their anaerobic (low oxygen) environment, leaf and wood litter decompose slowly, allowing carbon to remain stored for centuries or millennia (Howard et al., 2014). Mangrove forests store large amounts of carbon in their biomass and sediments and have the highest carbon density of any forest type (Adame et al., 2021; Donato et al., 2011). Their soils can hold up to three times more carbon per hectare than tropical upland forests (Dahdouh‐Guebas, 2011; Pendleton et al., 2012).

HMs, such as Cd, Pb, and Hg, can hinder mangrove growth and photosynthesis, thereby reducing biomass and carbon storage (Alongi, 2020). They can alter sediment microbial communities, disrupting decomposition and carbon cycling (Zeng et al., 2024), and increase organic matter breakdown by destabilizing sediments, potentially releasing stored carbon into the atmosphere (Enya et al., 2020). Overall, contamination undermines mangroves’ role in mitigating climate change through blue carbon sequestration (Analuddin et al., 2015).

This study investigated the relationship between HM contamination and carbon stocks in Puerto Rican mangrove ecosystems. Building on previous work that quantified hurricane‐related damage and carbon storage using remote sensing in northeastern and southwestern sites (Howe et al., 2025), we focused on sediment contamination and its potential influence on carbon cycling. Specifically, we aimed to (1) quantify the extent of HM contamination in mangroves subjected to different levels of anthropogenic impact, and (2) assess whether contamination levels are associated with changes in sediment carbon stocks.

## 2 MATERIALS AND METHODS

### 2.1 Study area

**FIGURE 1:**
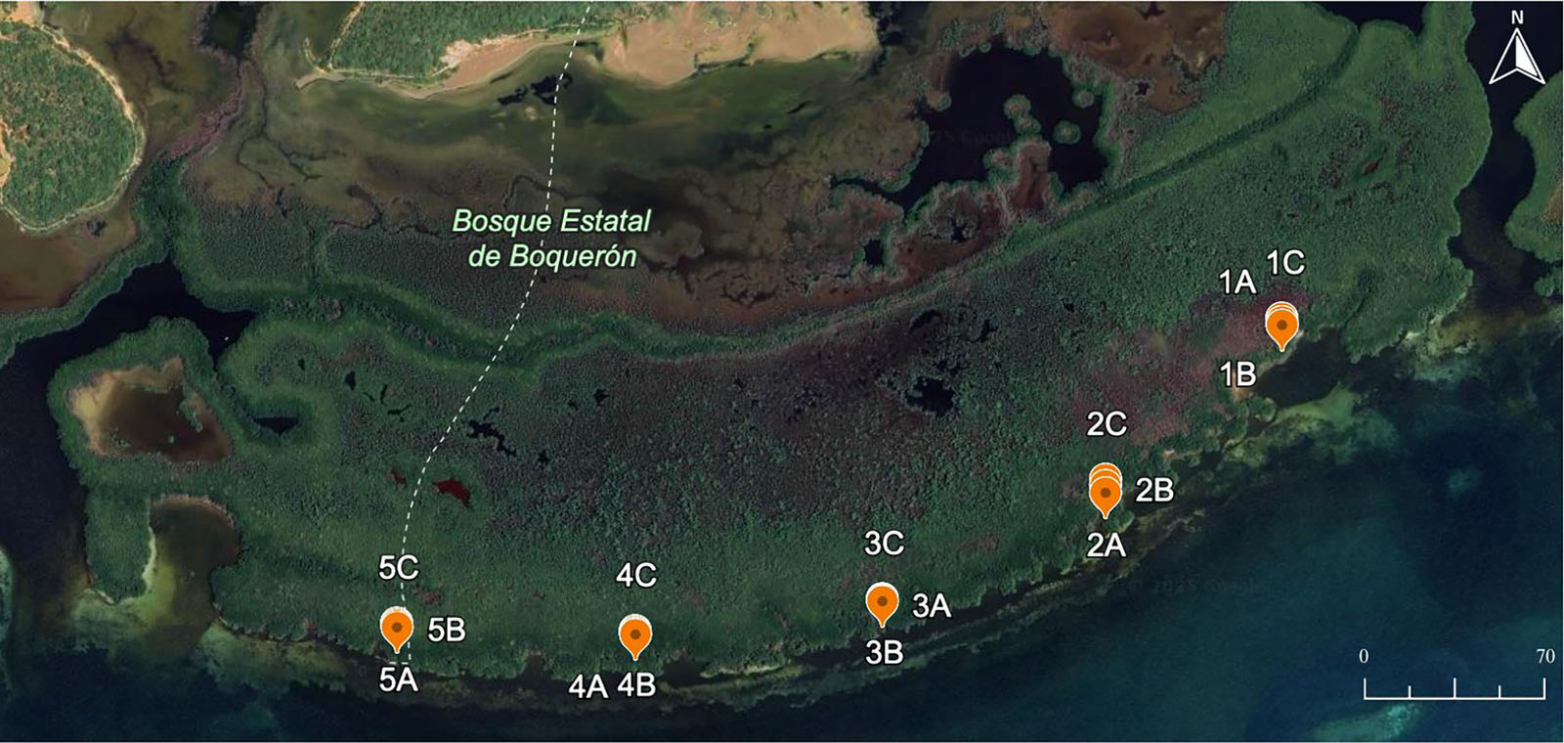
Map of the La Parguera mangrove study site in southwest Puerto Rico showing locations for heavy metal sampling. Imagery from Google Earth using Landsat 8 operated by NASA and USGS.

**FIGURE 2:**
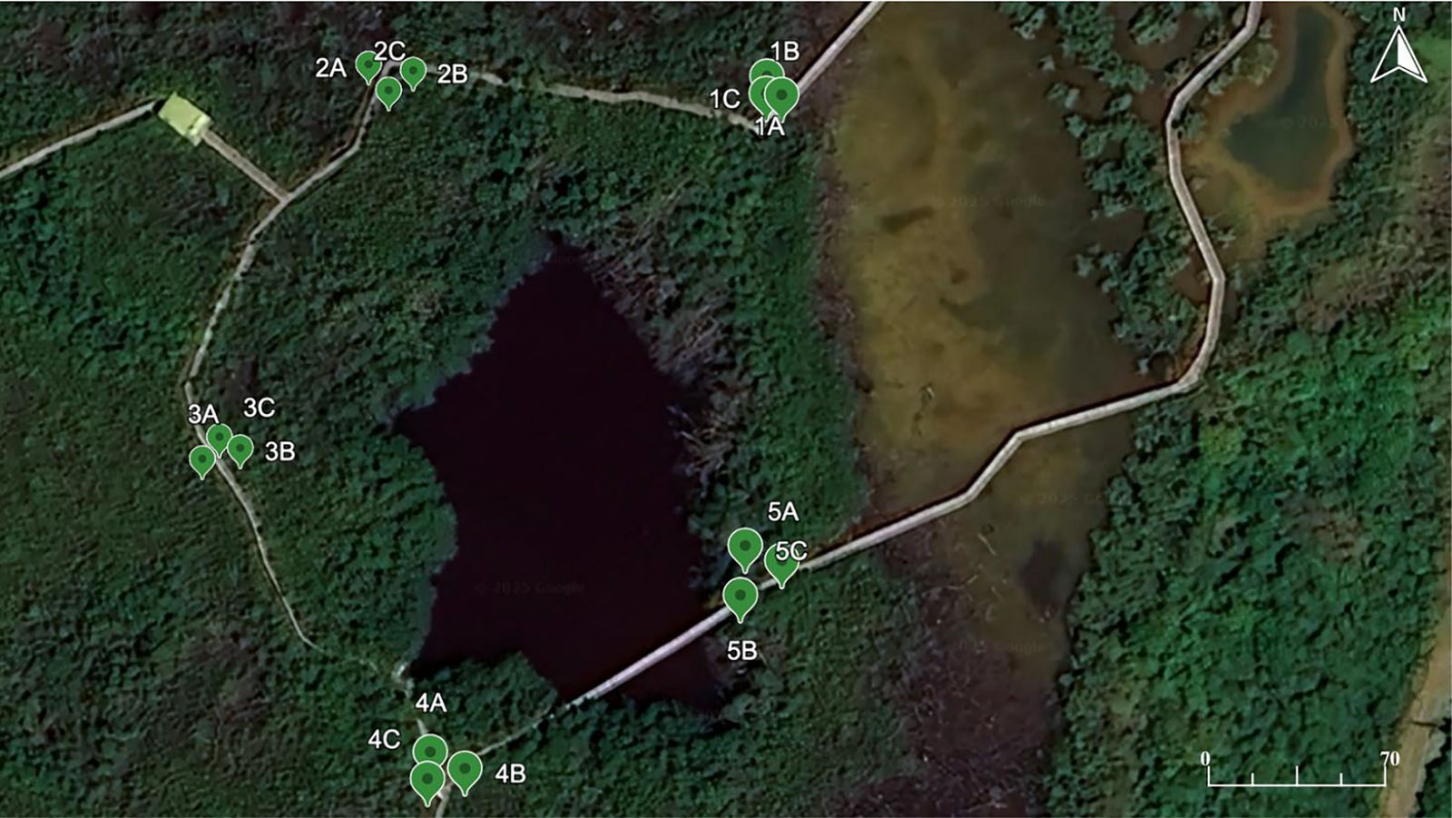
Map of the Laguna Grande mangrove study site in Fajardo, Puerto Rico, showing locations for metal sampling. Imagery from Google Earth using Landsat 8 operated by NASA and USGS.

The research focused on two study areas: La Parguera (Figure 1) and Laguna Grande (Figure 2). The La Parguera site is located in southwest Puerto Rico, while the Laguna Grande site is situated in the northeast. The study area in La Parguera encompasses the Boquerón State Forest and the La Parguera Natural Reserve, which are managed by the Puerto Rico Department of Natural and Environmental Resources (DNER) (Hernández et al., 2021).

### 2.2 Sediment sampling

Sediment sampling was conducted in two mangrove ecosystems: La Parguera and Laguna Grande, Puerto Rico. In La Parguera, the mangrove forest was divided into five subsites ∼410 m apart, spanning the full extent of the main mangrove island. At each subsite, three sediment cores were collected along a transect perpendicular to the shoreline, with cores spaced approximately 9 m apart from the coastline moving inland, totaling 15 cores. In Laguna Grande, five sampling sites were selected along a 100 km stretch of the mangrove forest edge. At each site, three sediment cores were collected from randomly selected points approximately 10 m apart, alsototaling 15 cores.

Sediment cores were collected using a Wildco Hand Core Sediment Sampler (Product No. 243‐G31), which features a clear polycarbonate barrel with a 7.6‐cm (3 in.) internal diameter and a maximum sampling depth of 50 cm. At each location, surface sediments (0–0.5 m) were extracted and retained in the sampling tubes. Samples were kept on ice in coolers until they were transported to laboratory facilities.

Sampling at La Parguera was completed in June 2023, and the samples were transported to the NOAA National Marine Fisheries Service James J. Howard Marine Laboratory, Sandy Hook, NJ, where they were sectioned into 7‐cm intervals, yielding 35 composite samples across all cores. Samples were oven‐dried at 60°C, stored in their sterile glass containers, and sent to the UMass Soil and Plant Tissue Testing Lab (Amherst, MA) for HM analysis.

**Core Ideas**

- Metal levels vary due to anthropogenic inputs (Laguna Grande) and tidal flushing (La Parguera).
- This study broadens known metal concentration ranges in mangrove sediments for future research.
- Metal‐carbon correlations differ by site, serving as indicators of metal sources and conditions.
- Metal retranslocation and bioavailability depend on type and site conditions, posing potential risks.
- Site‐specific factors regulate metals, highlighting the need for targeted mangrove management strategies.

Sampling at Laguna Grande took place in June 2024. Cores were transported to the USGS Woods Hole Coastal and Marine Science Center (Woods Hole, MA), where each of the cores was sectioned into 1‐cm intervals and sampled every 12 cm to produce 35 representative samples. These samples were freeze‐dried, sealed in polyethylene bags, and also submitted to the UMASS Soil and Plant Tissue Testing Lab for analysis.

This sampling design and processing approach aligns with methods used in previous HM assessments in mangrove ecosystems (i.e., Analuddin et al., 2017; Analuddin et al., 2023).

### 2.3 Leaf samples

Green (GL) and senescent leaves (SL) were collected from *Rhizophora mangle* (red mangrove) trees, the dominant species at both La Parguera and Laguna Grande. At each subsite, leaf samples were collected from both young and mature trees to capture potential age‐related differences in trace metal accumulation. GL and senescent leaves were selected from the same individual trees whenever possible, and these were clipped from similar positions along small twigs near the outer canopy to ensure consistency in leaf age and exposure. All leaves were placed in plastic bags and stored in a cooler until transported to the USGS Woods Hole Coastal and Marine Science Center. Upon arrival, the leaves were oven‐dried and sealed in clean plastic bags before being shipped to the UMASS Soil and Plant Tissue Testing Lab for HM analysis. A total of 100 leaf samples (50 senescent and 50 green) were collected from each site, resulting in 200 samples overall.

### 2.4 Chemical analysis

HM concentrations in soil and plant tissue samples were analyzed by the UMASS Soil and Plant Tissue Testing Lab using EPA method 3050B for acid digestion of sediments and method 6010D for subsequent elemental analysis via inductively coupled plasma‐optical emissions spectrometry (U.S. EPA, 1996, 2014). Samples were digested using concentrated hydrochloric and nitric acid with a hydrogen‐peroxide addition, following EPA guidelines. Sediment samples containing shell fragments were pretreated with 2.5 mL of dilute nitric acid and incubated for 12 h to remove excess calcium carbonate prior to digestion. These methods have been widely used in environmental studies for determining trace metal concentrations in both soils and plant tissues (Analuddin et al., 2017; Fonseca et al., 2013).

### 2.5 Empirical equations

We used the empirical equations from Maldonado‐Román et al. (2016) to calculate retranslocation percent (RT%) and bioconcentration factors (BCFs). The RT% is derived from differences in HM concentrations between senescent (yellow) and GL. The BCF is derived from differences in HM concentrations in soil and leaf tissue (Hammad, 2011; Maldonado‐Román et al., 2016; Mellem et al., 2009) as follows:

(1) $$\mathrm{RT}\%=\left(1-\frac{\left[\mathrm{HM}\;\mathrm{senecent}\right]}{\mathrm{HM}\;\mathrm{green}}\right)\times 100$$

(2) $$\mathrm{BCF}=\left(\frac{\mathrm{HM}\;\mathrm{leaves}}{\mathrm{HM}\;\mathrm{soil}}\right)$$

### 2.6 Statistical analysis

Differences between sites and subsites were evaluated with a two‐way analysis of variance with site and subsite as the main effects. Relationships between metals and soil carbon were explored with Pearson (linear) and Spearman (non‐linear) correlations.

## 3 RESULTS

### 3.1 HM concentrations

#### 3.1.1 Soils

Metal concentrations differed significantly (*p* < 0001) between La Parguera and Laguna Grande soils for all metals analyzed, with concentrations higher at the Laguna Grande site (Table 1). There was significant variation among subsites for some metals (Table 2). At the La Parguera site, subsite 1 had significantly (*p* < 0.05) higher levels of arsenic (As) and copper (Cu) than the other subsite, and subsite 3 had higher levels of chromium than most of the other subsites. At the Laguna Grande site, there were occasional significant subsite differences, but no consistent patterns across different metals.

**TABLE 1:** Levels (ppm) of arsenic, cadmium, chromium, copper, nickel, lead, and zinc in mangrove soils sampled at two sites.

Metal	La Parguera (*n* = 34)			Laguna Grande (*n* = 35)	
	Mean	S.E.		Mean	S.E.
Arsenic	3.34	0.28	***	15.21	1.96
Cadmium	0	0	***	0.41	0.07
Chromium	4.49	0.48	***	36.24	7.42
Copper	7.53	2.11	***	55.98	9.8
Nickel	4.51	0.43	***	8.57	0.63
Lead	1.19	0.07	***	4.28	0.55
Zinc	5.12	0.44	***	54.9	11.23

*Note*: Values are mean and standard error (S.E.) of samples taken at five subsites and three depths at each site.

*** indicates that sites are significantly different at *p* < 0.001.

**TABLE 2:** Levels (ppm) of arsenic, cadmium, chromium, copper, nickel, lead, and zinc in mangrove soils at five subsites at two sites in Puerto Rico.

			Arsenic		Cadmium		Chromium		Copper		Nickel		Lead		Zinc	
Site	Subsite	N	Mean	S.E.	Mean	S.E.	Mean	S.E.	Mean	S.E.	Mean	S.E.	Mean	S.E.	Mean	S.E.
La Parguera	1	3	7.2a	1.4	0.0a	0.0	3.6bc	2.2	29.2a	23.4	3.6bc	2.2	1.3a	0.3	4.6ab	2.2
	2	8	2.6b	0.3	0.0a	0.0	2.0c	0.3	4.2b	1.0	2.4c	0.2	1.1a	0.1	4.2ab	0.9
	3	8	3.0b	0.3	0.0a	0.0	6.7a	0.8	7.56b	0.8	5.4ab	0.5	1.1a	0.1	4.0b	0.5
	4	6	2.7b	0.2	0.0a	0.0	3.2bc	0.8	3.6b	0.3	3.5c	0.9	1.0a	0.1	5.0ab	0.9
	5	9	3.5b	0.4	0.0a	0.0	5.9ab	0.9	5.9b	0.6	6.7a	0.9	1.4a	0.2	7.2a	0.9
Laguna Grande	1	7	13.3a	1.4	0.9a	0.1	22.9a	0.7	35.4a	2.0	9.3b	0.7	2.2b	0.2	31.6a	3.6
	2	8	17.9a	4.8	0.1b	0.1	65.6a	26.4	84.3a	36.1	7.4bc	1.6	3.4ab	0.4	91.8a	44.1
	3	8	9.6a	0.9	0.0b	0.0	29.0a	3.8	33.2a	8.7	5.3c	0.8	6.8a	1.9	37.2a	10.9
	4	5	22.0a	2.9	0.6a	0.2	29.8a	4.4	48.1a	7.3	13.4a	1.3	4.8ab	0.5	50.1a	6.9
	5	7	15.7a	7.6	0.6a	0.1	40.6a	18.6	75.9a	21.4	9.5b	0.5	4.1ab	1.2	59.6a	19.3

*Note*: Values are mean and standard error (S.E.) of three to nine samples taken over three depths at each subsite. Subsite means followed by different letters within a site are significantly different at *p* < 0.05.

#### 3.1.2 Leaves

**FIGURE 3:**
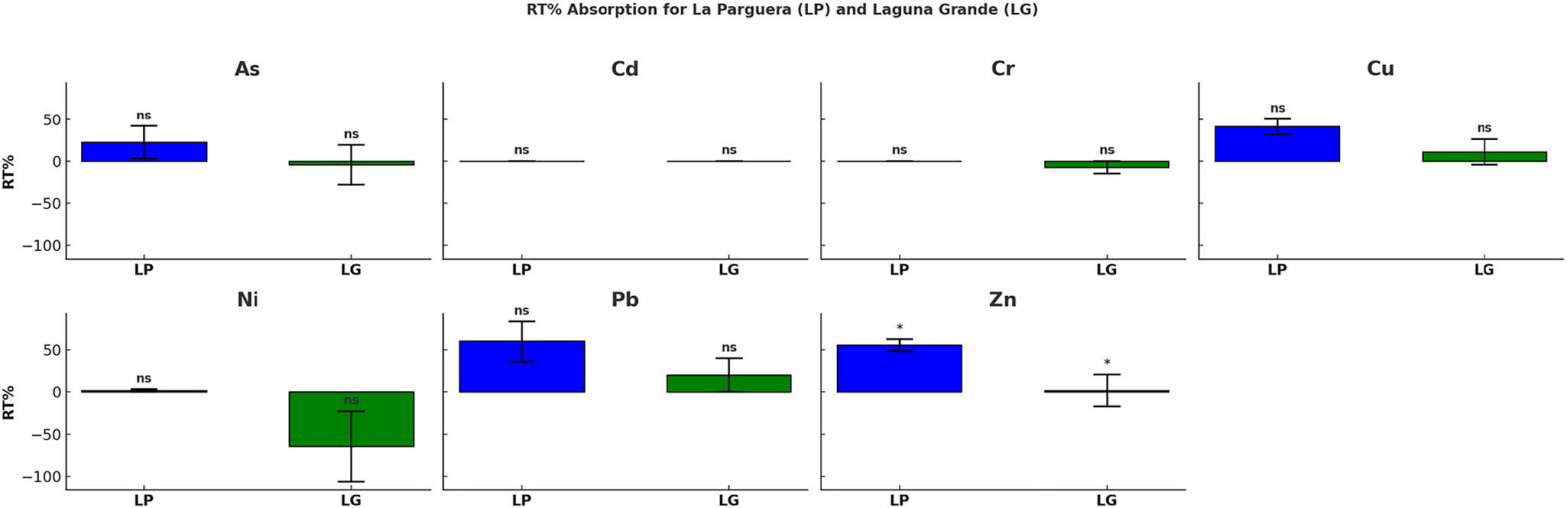
Relative translocation percentage (RT%) of heavy metals (As, Cd, Cr, Cu, Ni, Pb, and Zn) in mangroves at the La Parguera (LP) and Laguna Grande (LG) sites. Bars represent mean RT% values for each site, with standard error. There was a statistically significant (*p* < 0.05) difference between sites only for zinc (Zn). ns = not significant, while the asterisk (*) represents a significant relationship.

**FIGURE 4:**
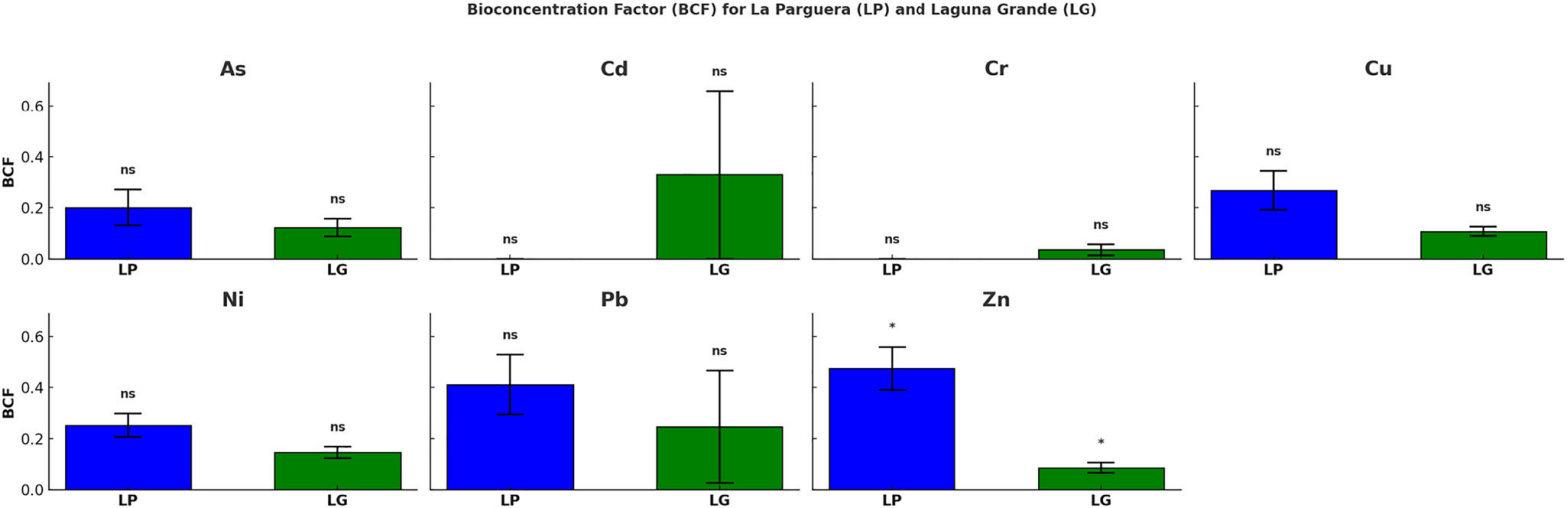
Bioconcentration factors (BCFs) of heavy metals (As, Cd, Cr, Cu, Ni, Pb, and Zn) in mangroves at the La Parguera (LP) and Laguna Grande (LG) sites. Bars represent mean BCF values for each site with standard error. There was a statistically significant (*p* < 0.05) difference between sites only for zinc (Zn). ns = not significant, while the asterisk (*) represents a significant relationship.

Mean concentrations of HMs in mangrove leaves were generally higher at the Laguna Grande site compared to La Parguera (Table 3). Relative translocation percentages (RT%), which represent the redistribution of metals between GL and senescent leaves (Figure 3), were generally similar across sites. However, RT% for Zn was significantly higher at La Parguera (*p* < 0.05). The BCF, calculated as the ratio of metal concentrations in leaf tissue to those in sediments (Figure 4), also showed higher values at La Parguera for most metals, though only Zn was significantly different between sites (*p* < 0.01).

**TABLE 3:** Levels (ppm) of arsenic, cadmium, chromium, copper, nickel, lead, and zinc in live (green) and senescent (yellow) mangrove leaves were sampled at two sites.

La Parguera					Laguna Grande				
Metal	Yellow		Green			Yellow		Green	
	Mean	S.E.	Mean	S.E.		Mean	S.E.	Mean	S.E.
Arsenic	0.55	0.23	0.76	0.19	*	1.81	0.29	1.54	0.50
Cadmium	0.00	0.00	0.00	0.00		0.00	0.00	0.00	0.00
Chromium	0.00	0.00	0.00	0.00	ns	1.16	0.54	0.39	0.39
Copper	1.12	0.17	1.92	0.02	***	4.36	0.73	4.96	0.43
Lead					ns	1.58	0.41	0.96	0.01
Nickel	0.94	0.02	0.96	0.01	ns	0.99	0.99	0.18	0.18
Zinc	0.19	0.19	0.76	0.19	**	3.98	0.78	4.02	0.22

*Note*: Values are mean and standard error (S.E.) of samples taken at five subsites at each site. There were no significant differences between green and yellow leaves for any metal at either site.

*p < 0.5, **p < 0.01, and ***p < 0.001 indicate that sites are significantly different, and “ns” indicates no significant difference between sites.

### 3.2 HM versus blue carbon

**FIGURE 5:**
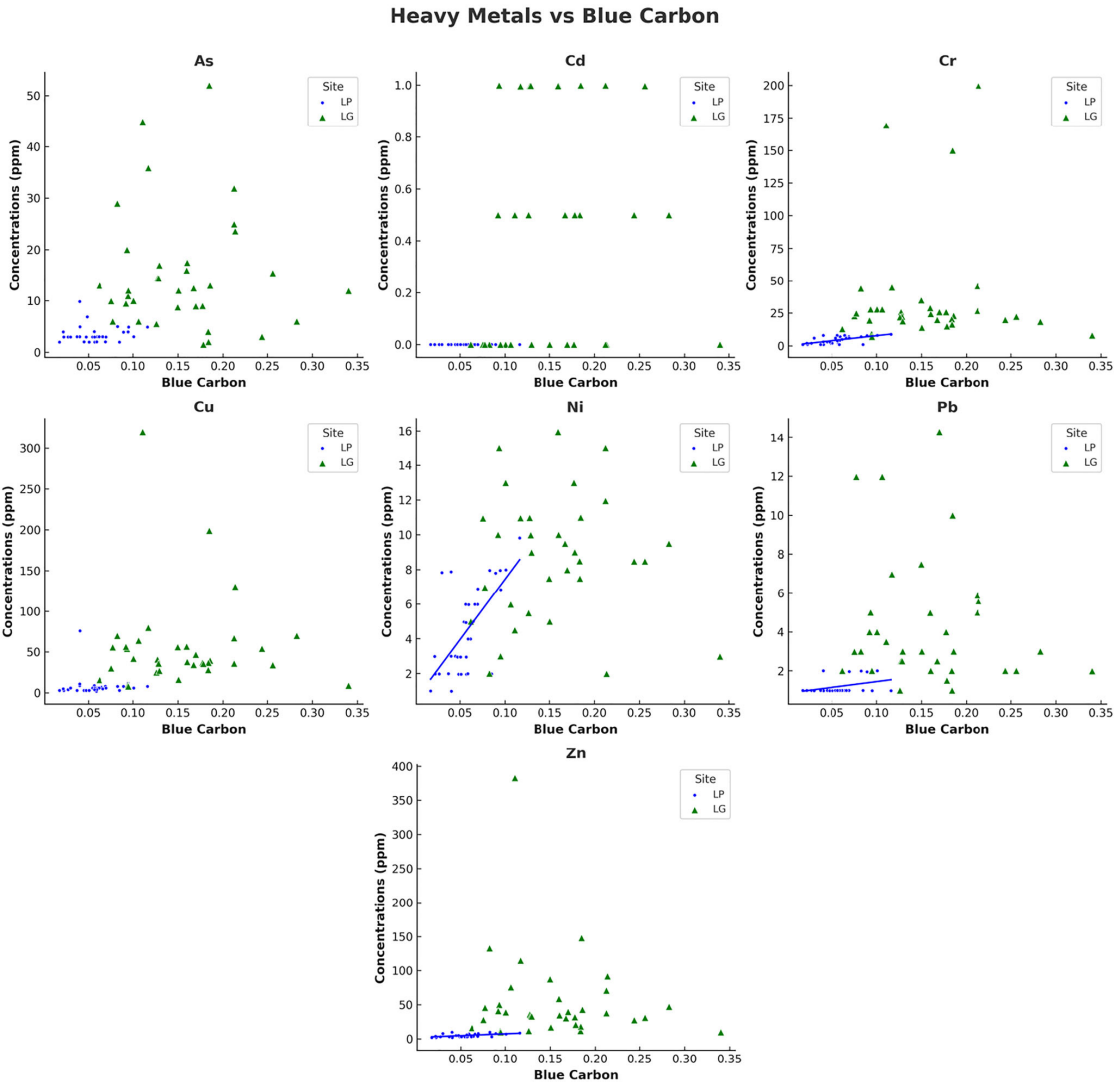
Relationships between heavy metal concentrations (ppm) and soil carbon concentration (%) across two locations: Laguna Grande (green) and La Parguera (blue). Each panel represents a heavy metal (As, Cd, Cr, Cu, Ni, Pb, and Zn). Scatter points denote measured data, while solid lines indicate statistically significant (*p* < 0.05, *p* < 0.01, *p* < 0.001) linear regression fits. Nonsignificant relationships do not include a regression line.

**FIGURE 6:**
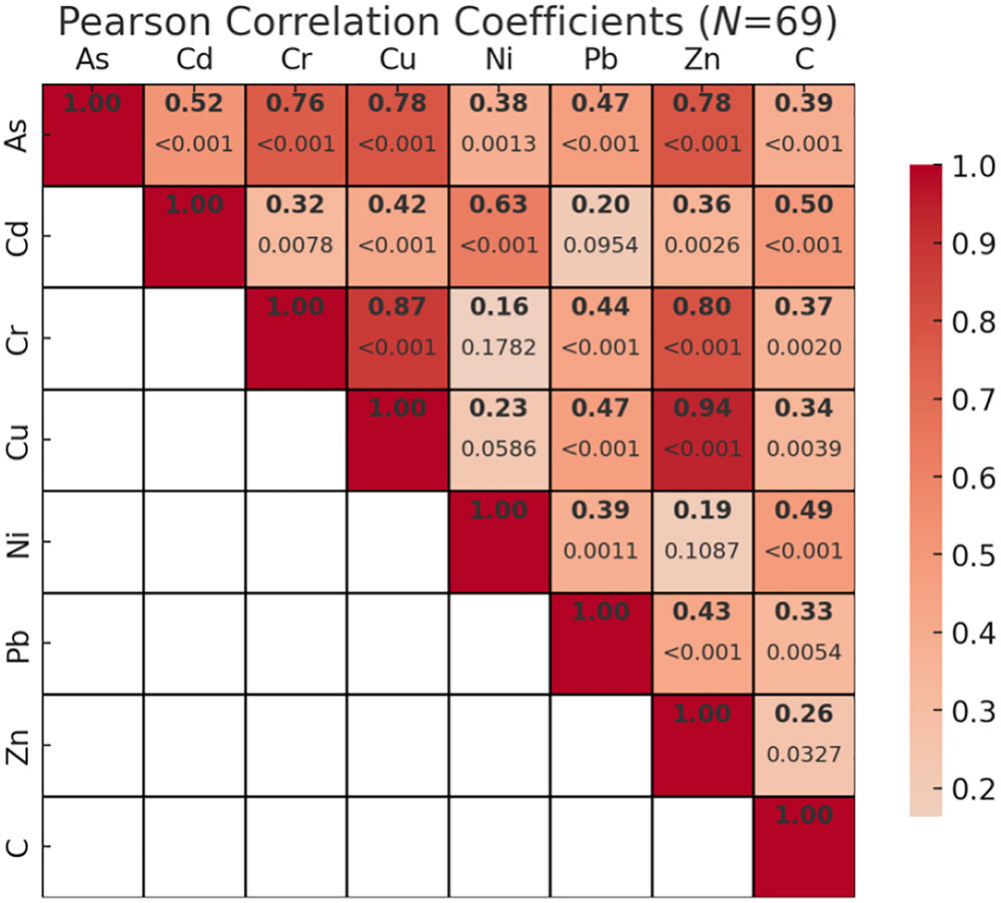
Pearson correlation coefficients, with *p*‐values underneath, for heavy metal concentrations and blue carbon storage across all samples from both mangrove sites (*N* = 69).

**FIGURE 7:**
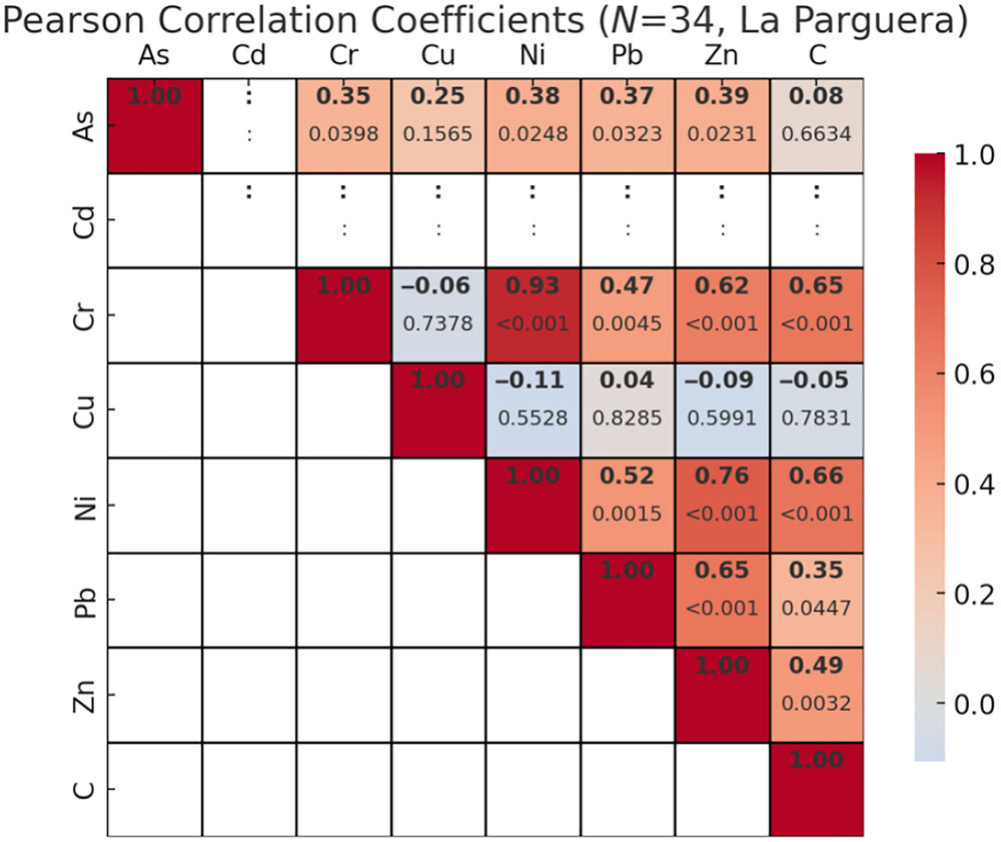
Pearson correlation coefficients, with *p*‐values underneath, for heavy metal concentrations and blue carbon storage for samples from the La Parguera site (*N* = 34).

**FIGURE 8:**
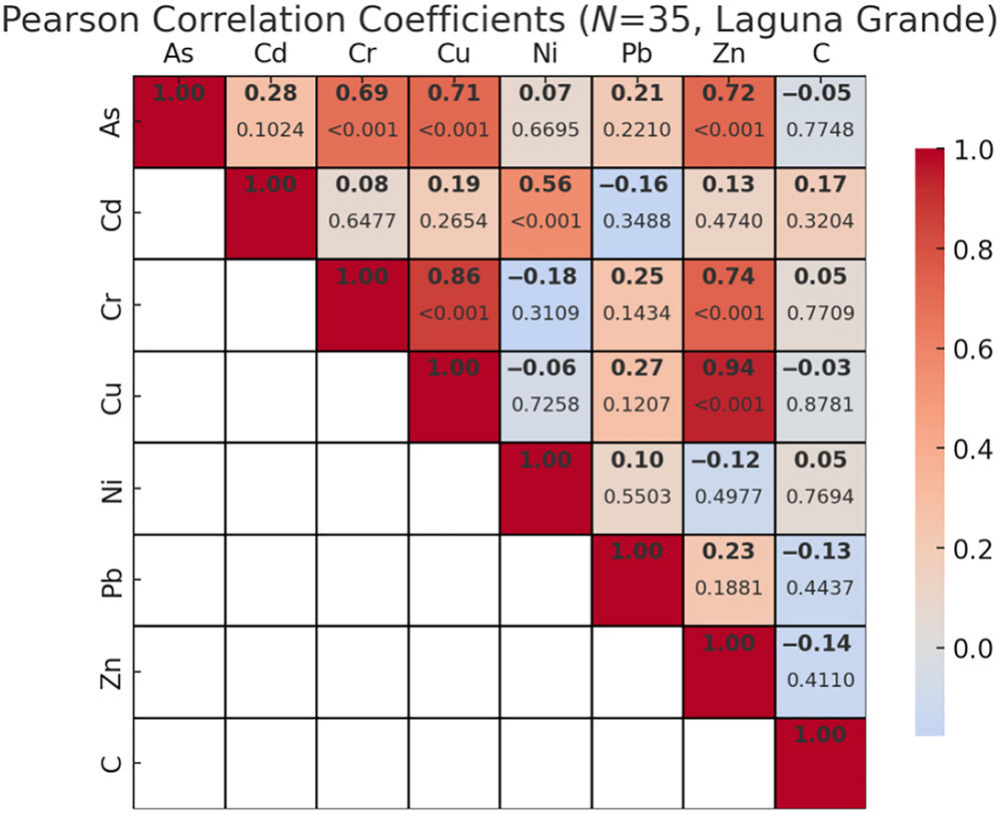
Pearson correlation coefficients, with *p*‐values underneath, for heavy metal concentrations and blue carbon storage for samples from the Laguna Grande site (*N* = 35).

Strong positive correlations were found between total sediment carbon content and concentrations of most HMs across both sites (Figure 5), and among several metals themselves (Figure 6). These patterns were primarily driven by the higher concentrations of both metals and carbon at the Laguna Grande site. When analyzed by site, distinct differences emerged: at La Parguera (Figure 7), chromium (Cr), nickel (Ni), Pb, and Zn were significantly correlated with carbon (*p* < 0.05), while Cu showed no significant correlation. In contrast, at Laguna Grande (Figure 8), no significant correlations were found between carbon and any metal, and correlations among metals were weak. This study focused on surface sediment samples, and depth‐resolved analyses were not conducted.

The significant positive correlations among several metals in La Parguera (Figure 7), particularly Cr, Ni, Pb, and Zn, may suggest similar geochemical behavior or a shared contamination source, such as runoff or historical anthropogenic inputs. These metals may bind to similar organic fractions or particle sizes within the sediment, contributing to their co‐accumulation. In contrast, the weak correlations among metals at Laguna Grande (Figure 8) may reflect more diffuse or heterogeneous metal sources, different sediment characteristics, or site‐specific redox conditions that influence metal mobility and retranslocation. Understanding these correlations provides insight into how mangrove sediments process and store HMs under varying ecological and anthropogenic conditions.

## 4 DISCUSSION

### 4.1 Patterns of HM contamination across sites

Background levels of HMs in pristine mangrove sites are generally low and mirror the local sediment geochemical baseline (Mohammed et al., 2024). Global averages—such as those published by Turekian and Wedepohl (1961)—are often used as reference points, with values of 25 ppm for Cu, 70 ppm for Zn, 17 ppm for Pb, 100 ppm for Cr, 45 ppm for Ni, and around 0.3 ppm for Cd. These values are corroborated by region‐specific studies. For example, Majumdar et al. (2023) observed Cu levels between 15 and 25 ppm, Zn between 50 and 70 ppm, and Pb around 10–15 ppm in the Indian Sundarbans, suggesting minimal anthropogenic input. Alongi (2017) noted that undisturbed mangroves in various regions—including the Caribbean—exhibit metal concentrations below global averages.

In this study, HM concentrations in surface sediments were relatively low at both Puerto Rican sites (Table 2), suggesting low baseline values in the region. However, metals were consistently lower at La Parguera than at Laguna Grande. La Parguera's isolation from urban centers and its location within a marine protected area with regular tidal flushing may contribute to its lower contamination levels. In contrast, Laguna Grande is located ∼55 km from San Juan and adjacent to Fajardo, a growing urban center. Anthropogenic sources likely influencing Laguna Grande include urban runoff, roadways, septic discharge, marina infrastructure, and recreational boating. Similar urban‐associated inputs have been documented in other Caribbean mangrove ecosystems (Maldonado‐Román et al., 2016).

Metal concentration trends also varied by metal. For instance, Zn and Cr were significantly higher at Laguna Grande than La Parguera (*p* < 0.05), while Pb and Ni showed less pronounced differences (Table 2). The low concentrations across both sites, despite these differences, suggest minimal large‐scale industrial contamination. Figure 6 illustrates inter‐metal correlations that provide further insight into possible shared sources. For example, Cr and Ni were significantly correlated at La Parguera but not at Laguna Grande, suggesting a natural geogenic origin at the former and more diffuse or unrelated sources at the latter. These patterns are consistent with site‐level differences in land use, hydrology, and geochemistry.

### 4.2 Effects of HM contamination on carbon cycling and plant uptake

HMs such as Cu, Zn, and Pb are known to interact with organic matter through adsorption and complexation, especially in mangrove sediments rich in detritus and microbial biomass (Marchand et al., 2006; Turekian & Wedepohl, 1961). These interactions typically produce strong positive correlations between metal concentrations and sediment carbon content, particularly in organic‐rich surface layers. Figure 5 shows these relationships across both sites. Interestingly, the strong correlations observed at the regional scale are largely driven by the elevated metal and carbon concentrations at Laguna Grande. However, within‐site correlations differ: La Parguera exhibits strong positive correlations between carbon and Cr, Ni, Pb, and Zn (Figure 7), while no such relationship was observed at Laguna Grande (Figure 8).

These differences suggest distinct underlying mechanisms. At La Parguera, plant litter inputs and organic‐rich sediment likely enhance metal retranslocation, leading to co‐accumulation of metals and carbon. In contrast, at Laguna Grande, external metal loading may overwhelm these natural retranslocation mechanisms, decoupling the carbon–metal relationship.

Biological uptake of metals was also explored using BCF and Rt% (Figures 3 and 4). BCF is a well‐established metric to assess plant metal accumulation. Following published thresholds (Baker et al., 2000; Yoon et al., 2006), plants with BCF > 1 are considered hyperaccumulators, BCF ≈ 1 indicates moderate accumulation, and BCF < 1 suggests low accumulation. In our study, Zn at La Parguera exhibited BCF values >1, suggesting moderate accumulation and values <1 suggest low accumulation. In our study, Zn at La Parguera exhibited BCF values > 1, indicating hyperaccumulation, while most other metals fell below this threshold.

RT% values revealed that Zn and Pb showed higher retranslocation in mangrove leaves at La Parguera, suggesting active uptake and possible internal storage mechanisms (Figure 3). Conversely, Cd and Ni exhibited lower RT% and higher BCF at Laguna Grande, suggesting high uptake potential but limited internal retranslocation, which may increase ecological risk. These results are consistent with previous studies that report metal mobility and plant exclusion strategies (Celis‐Hernández et al., 2022; Genchi et al., 2020).

High BCF and low RT% metals (i.e., Cd and Ni) are particularly concerning due to their mobility and bioavailability, which could result in phytotoxicity and downstream ecological effects. Differences in sediment characteristics, plant density, and species composition between the two sites may contribute to these patterns. Further research, including plant physiological assessments and metal speciation analysis, would help validate the observed relationships.

Overall, these findings indicate that while both mangrove ecosystems are relatively uncontaminated, their differing exposure to human activities and tidal connectivity strongly influence the accumulation and behavior of metals in sediments and plants, with implications for blue carbon stability and ecosystem health.

## 5 CONCLUSION

This study aimed to (1) quantify HM contamination across two mangrove ecosystems in Puerto Rico and (2) assess how this contamination relates to carbon stocks and cycling. Overall, HM concentrations were relatively low, but significant differences were observed between La Parguera and Laguna Grande. Elevated metal levels at Laguna Grande appear to result from anthropogenic sources, whereas lower levels at La Parguera may reflect tidal flushing and reduced urban influence.

Metal–carbon relationships varied markedly by site. At La Parguera, sediment carbon was significantly correlated with several metals (Cr, Ni, Zn, and Pb), indicating localized biogeochemical retranslocation. In contrast, Laguna Grande showed no such correlations despite higher concentrations of both metals and carbon, suggesting decoupling due to external inputs.

Plant–metal interactions also differed by site. La Parguera exhibited higher retranslocation (RT%) and BCF values for specific metals, particularly Zn, which is consistent with plant‐mediated stabilization. Laguna Grande, by contrast, exhibited high BCF and low RT% for toxic metals such as Cd and Ni, indicating greater mobility and potential ecological risk.

These findings underscore the importance of site‐specific factors—such as hydrology, land use, and vegetation—in shaping the behavior of HMs and their implications for blue carbon stability. Our results provide valuable reference points for future assessments of mangrove health and metal contamination across tropical coastlines.

## AUTHOR CONTRIBUTIONS

**Jahnelle Howe**: Conceptualization; data curation; formal analysis; funding acquisition; investigation; methodology; project administration; resources; software; visualization; writing—original draft; writing—review and editing. **Peter M. Groffman**: Conceptualization; investigation; methodology; project administration; supervision; validation; visualization; writing—review and editing. **William J. Hernández**: Writing—review and editing. **Shakila Merchant**: Writing—review and editing.

## CONFLICT OF INTEREST STATEMENT

The authors declare no conflicts of interest.

## Data Availability

The data supporting this study's findings is available in the Dryad data repository at [https://doi.org/10.5061/dryad.tx95×6b7g].
